# Supplementary material for: An injectable liposome-anchored teriparatide incorporated gallic acid-grafted gelatin hydrogel for osteoarthritis treatment
Source: Nat Commun. 2023 May 31;14:3159. doi: 10.1038/s41467-023-38597-0 (PMC10232438; doi:10.1038/s41467-023-38597-0)
Supplement: Supplementary file 1 — Supplementary Information [file 41467_2023_38597_MOESM1_ESM.pdf]

Point-of-care based design of liposome-anchored teriparatide incorporated gallic acid-grafted gelatin injectable hydrogel for osteoarthritis treatment

Supplementary Figures, Legends, and Tables

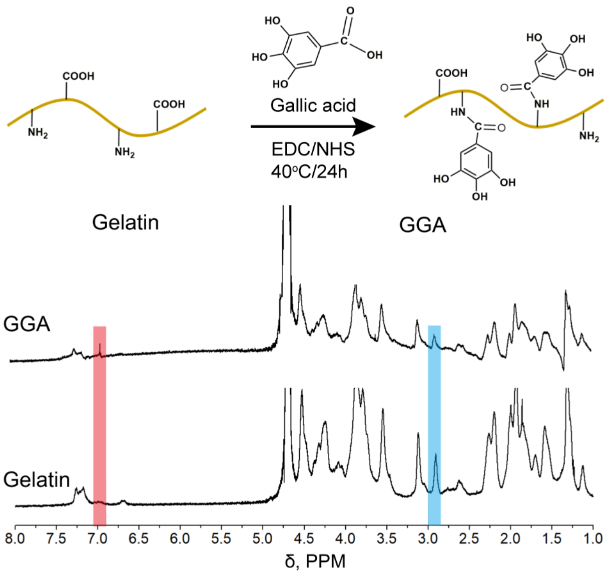

Supplementary Figure 1. The preparation process of gallic acid-grafted gelatin (GGA) and the ^{1}H Nuclear magnetic resonance spectroscopy of the GGA versus gelatin. EDC, N-(3-Dimethylaminopropyl)-N'-ethylcarbodiimide hydrochloride; NHS, N-Hydroxysuccinimide; GGA, gallic acid-grafted gelatin; PPM, partper million.

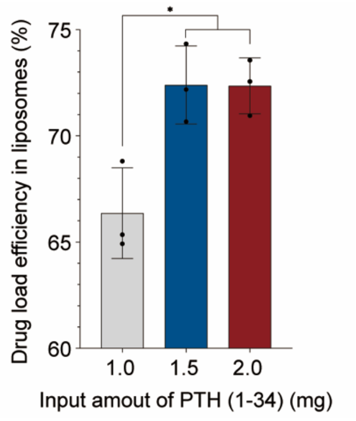

Supplementary Figure 2. The load efficiency of different PTH (1-34) input amount in the same amount of liposome. Data are presented as means ± SD of at least three replicate experiments.

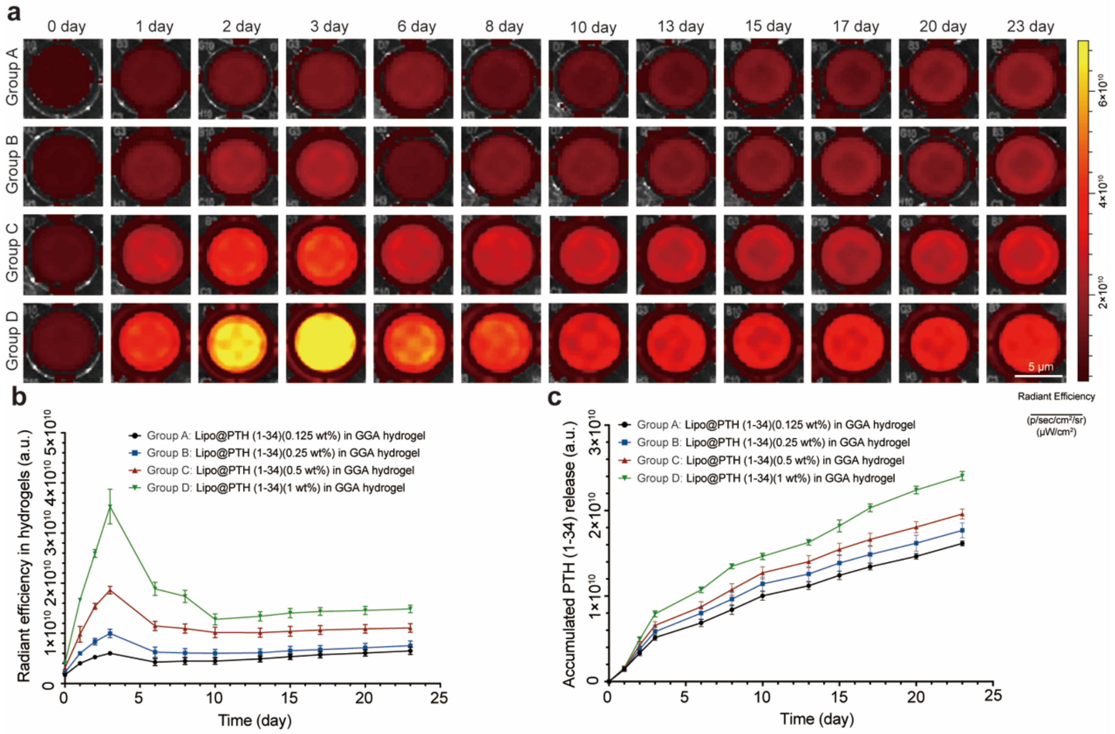

Supplementary Figure 3. The PTH (1-34) remained in hydrogels and its release profile during the in vitro immersion in PBS up to 23 days. **a** The IVIS images for the hydrogels. **b** The radiant efficiency in hydrogels. **c** The accumulated PTH (1-34) release profile calculated from immersion PBS solutions. Data are presented as means ± SD of at least five replicate experiments. A.u., arbitrary units.

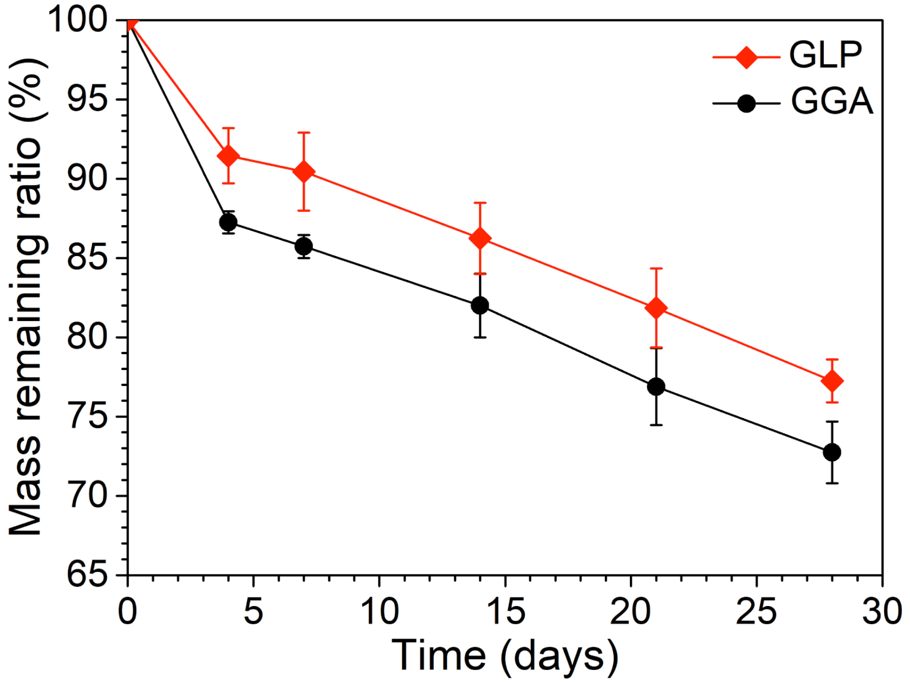

Supplementary Figure 4. The mass remaining ratio of the hydrogels immersed in PBS up to 28 days (37 ºC, rocking state). GGA, gallic acid-grafted gelatin; GLP, GGA@Lipo@PTH (1-34). Data are presented as means ± SD of at least three replicate experiments.

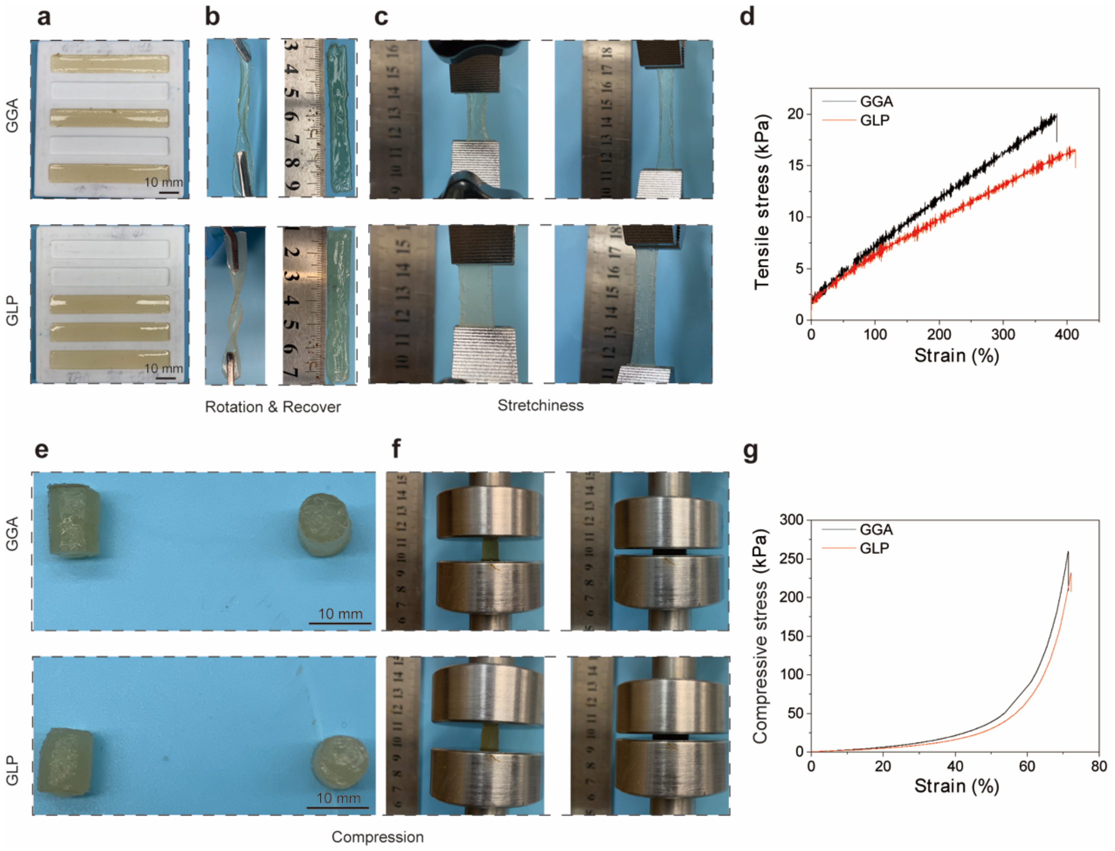

Supplementary Figure 5. The mechanical properties of the GLP hydrogel versus GGA hydrogel. **a** The fabrication of rectangular hydrogels in a mold. **b**, **c** The images for rotation and stretching tests. **d** The representative tensile stress versus strain curves. **e**, **f** The macroscopical images for the fabricated GGA, GLP hydrogels and the compression tests for these hydrogels. **g** The representative compressive stress versus strain curves. Data are presented as means ± SD of at least three replicate experiments. GGA, gallic acid-grafted gelatin; GLP, GGA@Lipo@PTH (1-34).

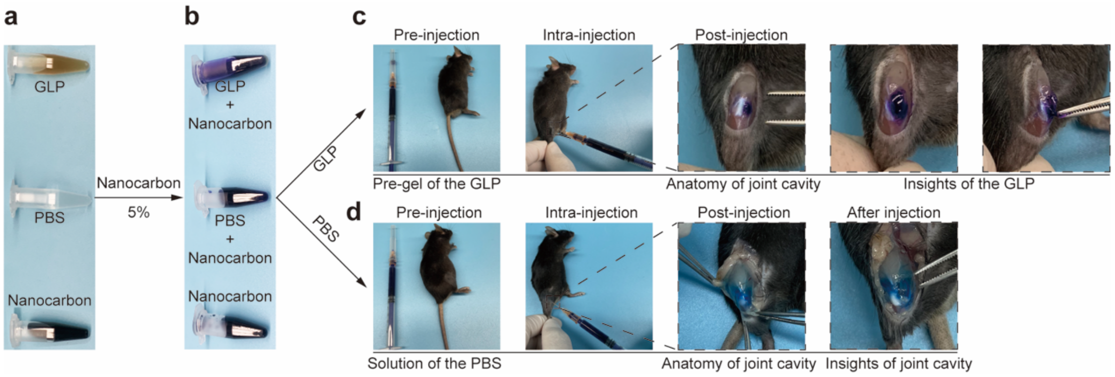

Supplementary Figure 6. The whole process for intra-articular injection with the GLP hydrogel versus PBS, demonstrating the in situ colloidal formation of the GLP hydrogel in the joint cavity after intra-articular injection. Data are presented as means ± SD of at least three replicate experiments. PBS, phosphate-buffered saline; GLP, GGA@Lipo@PTH (1-34).

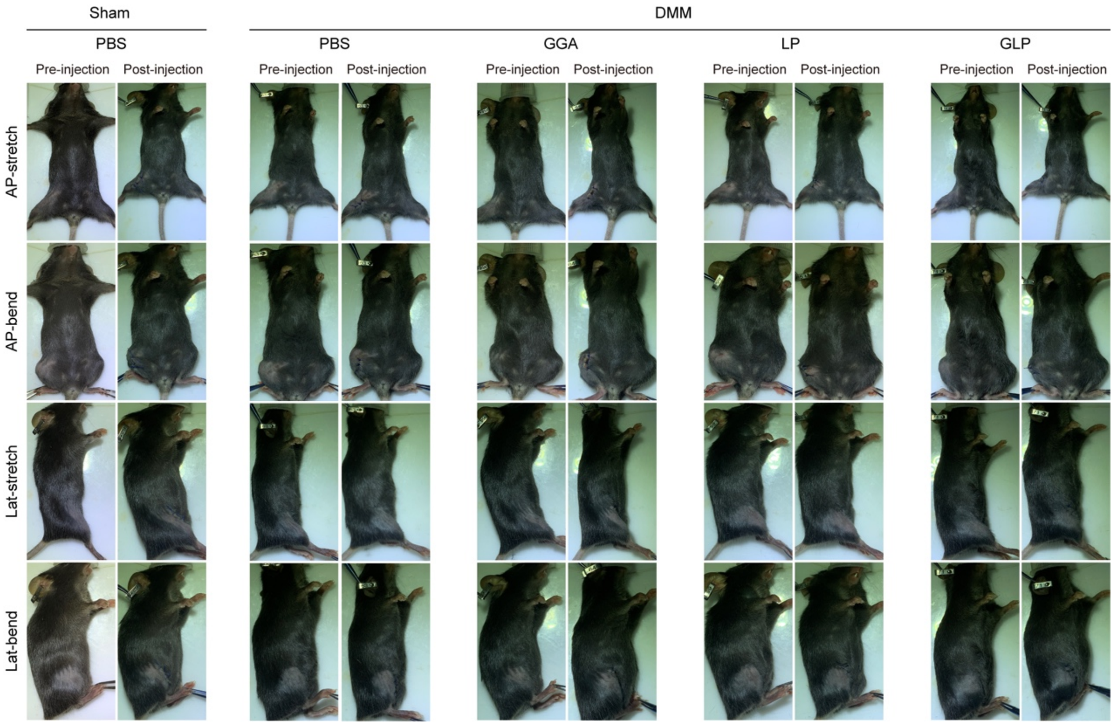

Supplementary Figure 7. The macrograph images of motion range for the mice after intra-articular injection with the hydrogels. Data are presented as means ± SD of at least three replicate experiments. Lat, lateral; AP, anteroposterior; DMM, destabilization of the medial meniscus; PBS, phosphate-buffered saline; GGA, gallic acid-grafted gelatin; LP, Lipo@PTH (1-34); GLP, GGA@Lipo@PTH (1-34).

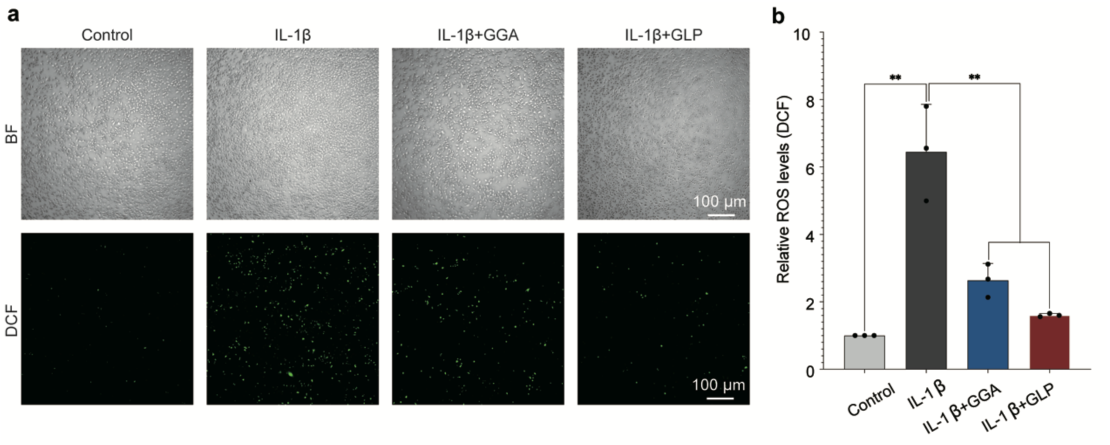

Supplementary Figure 8. The ROS-scavenging properties of the GLP and GGA hydrogels versus controls when co-culturing with the IL-1β-induced-chondrocytes by means of the DCFH-DA assay. Data are presented as means ± SD of at least three replicate experiments. Unpaired two-tailed *Student's t*-tests was used to calculate significant difference, $*p<0.05$, $**p<0.01$. BF, bright field; DCF, Dichlorodihydrofluorescein; GGA, gallic acid-grafted gelatin; GLP, GGA@Lipo@PTH (1-34).

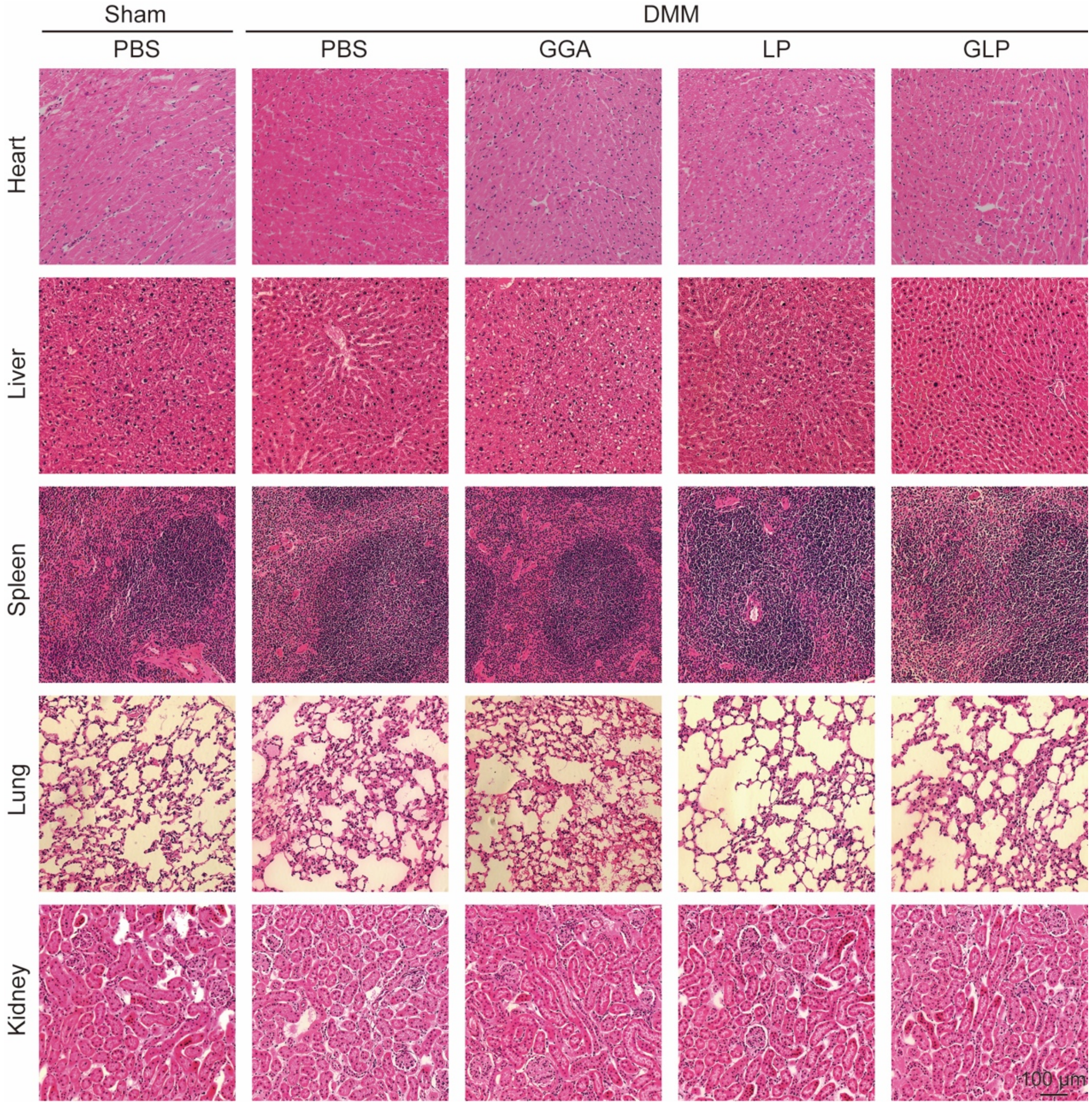

Supplementary Figure 9. H&E staining for the different organs in mice after intra-articular injection with different samples. Data are presented as means ± SD of eight replicate experiments. DMM, destabilization of the medial meniscus; PBS, phosphate-buffered saline; GGA, gallic acid-grafted gelatin; LP, Lipo@PTH (1-34); GLP, GGA@Lipo@PTH (1-34).

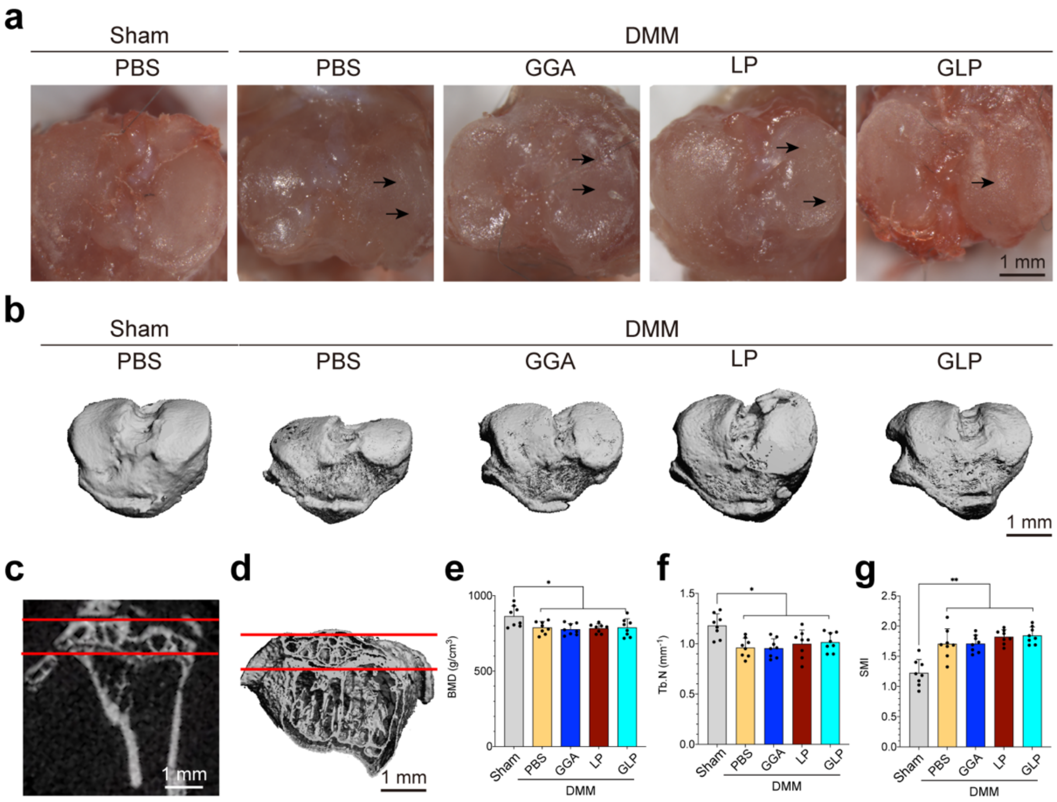

Supplementary Figure 10. The macrographic images and Micro-CT data for the tibial plateau of mice after intra-articular injection with different samples. **a** Macrographic images. The black arrows indicated the wear and rough areas in the surface of the articular cartilage. **b** Three dimensional images from Micro-CT detection. **c**, **d** The Micro-CT analysis region for the subchondral bone in tibial plateau. **e**, **f**, **g** The statistical bone-related parameters including BMD, Tb.N, and SMI from Micro-CT data. Data are presented as means ± SD of at least eight replicate experiments. Unpaired two-tailed *Student's t*-tests was used to calculate significant difference, *p<0.05, **p<0.01. DMM, destabilization of the medial meniscus; PBS, phosphate-buffered saline; GGA, gallic acid-grafted gelatin; LP, Lipo@PTH (1-34); GLP, GGA@Lipo@PTH (1-34); BMD, bone mineral density; Tb.N, trabecular number; SMI, structure model index.

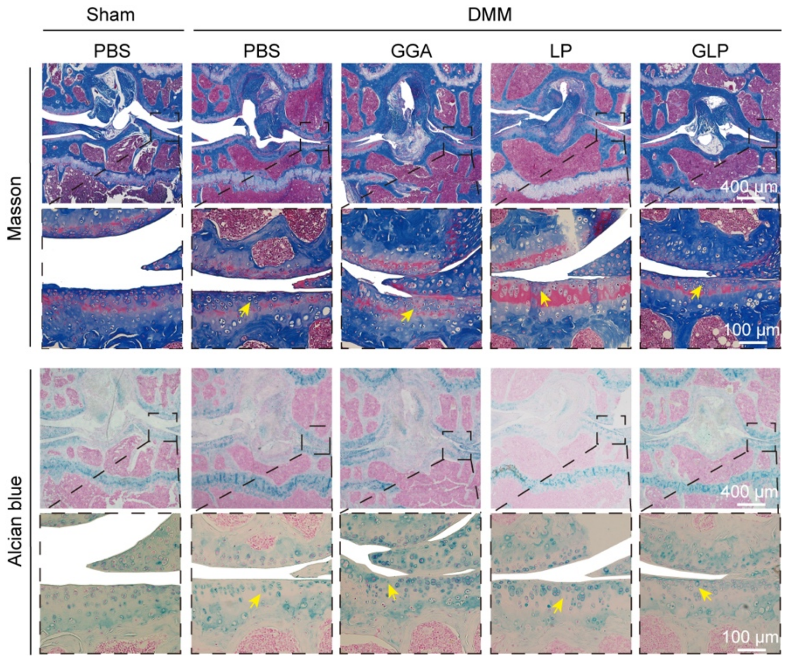

Supplementary Figure 11. **Masson and Alcian blue stainings of the arthrography section after intra-articular injection with different samples.** (n=8 independent experiments). The yellow arrows stand for the degenerated areas in the articular cartilage. DMM, destabilization of the medial meniscus; PBS, phosphate-buffered saline; GGA, gallic acid-grafted gelatin; LP, Lipo@PTH (1-34); GLP, GGA@Lipo@PTH (1-34).

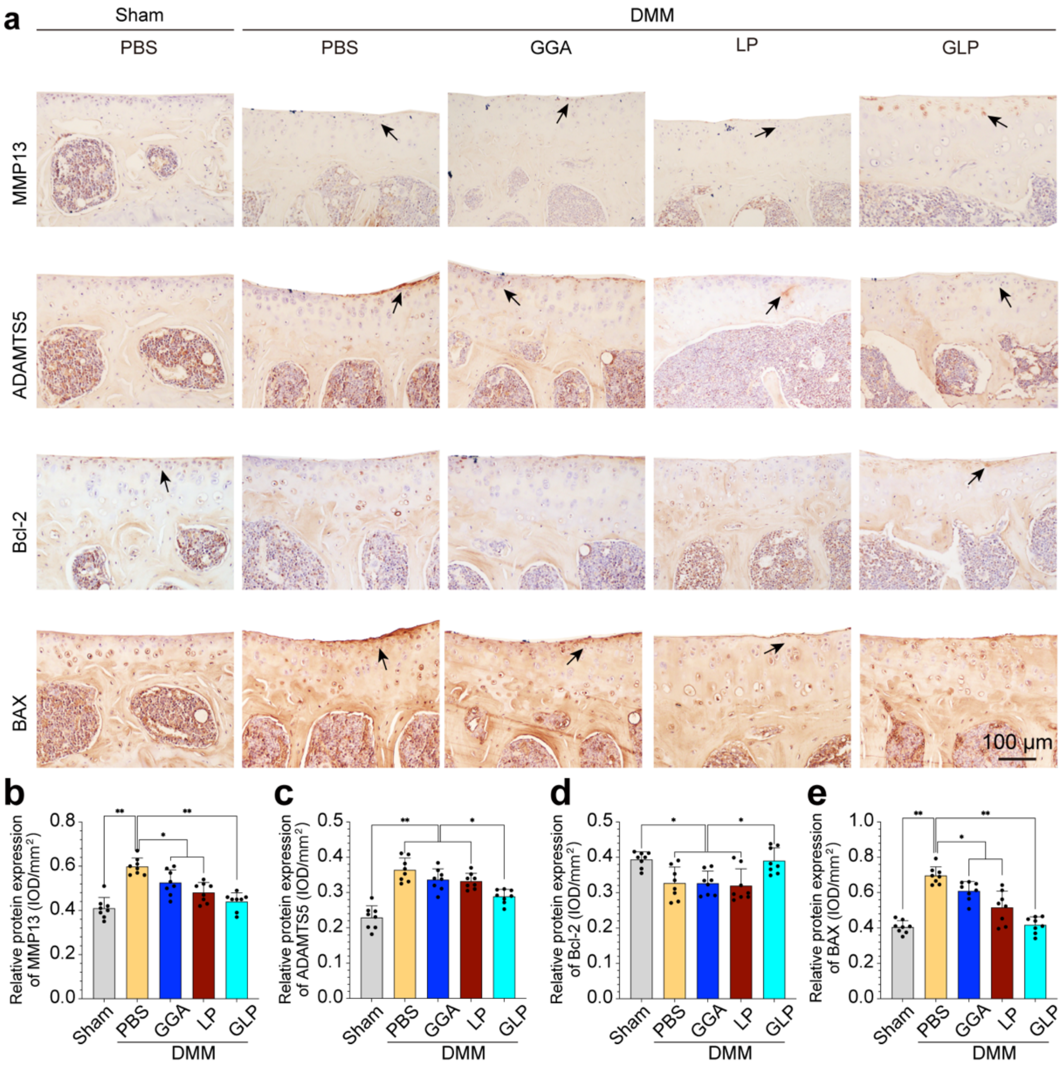

Supplementary Figure 12. Representative immunohistochemical staining for the ECM and chondrocytes of the arthrography section after intra-articular injection with different samples. **a** Immunohistochemical stained images for MMP13, ADAMTS5, Bcl-2, and BAX proteins. The black arrows indicate the immunohistochemical positively stained areas in the articular cartilage. **b**, **c**, **d**, **e** The statistical analysis results for those proteins expression from immunohistochemical stained images. Data are presented as means ± SD (n=8). Unpaired two-tailed *Student's t*-tests was used to calculate significant difference, *p<0.05, **p<0.01. DMM, destabilization of the

medial meniscus; PBS, phosphate-buffered saline; GGA, gallic acid-grafted gelatin; LP, Lipo@PTH (1-34); GLP, GGA@Lipo@PTH (1-34).

Supplementary Tables

Supplementary Table 1. Modified Mankin score principal.

Grading	Description
Score	Cartilage Erosion Scoring Criteria
0	Smooth non-eroded cartilage
1	Rough non-eroded cartilage
2	Superficial fibrillation
3	Separation of uncalcified from calcified cartilage
4	Erosion of uncalcified cartilage only
5	Erosion extending into calcified cartilage
6	Erosion down to subchondral bone
	Chondrocyte periphery staining
0	Normal
1	Slightly enhanced
2	Intensely enhanced
	Spatial arrangement of chondrocytes
0	Normal
1	Diffuse hypercellularity
2	Clustering
3	Hypocellularity
	Background staining intensity
0	Normal
1	Slight reduction
2	Moderate reduction
3	Severe reduction
4	No dye noted

Supplementary Table 2. The primers for RT-qPCR test.

Gene	NCBI Gene ID	GenBank Accession	Primer Sequence		Length
Gapdh	14447	NM_008085	Forward (5' -> 3')	TGTGTCCGTCGTGGATCTGA	20
			Reverse (5' -> 3')	TTGCTGTTGAAGTCGCAGGAG	21
Sox9	20682	NM_011448	Forward (5' -> 3')	CGGAACAGACTCACATCTCTCC	22
			Reverse (5' -> 3')	GCTTGCACGTCGGTTTTGG	19
Col2a1	12824	NM_031163	Forward (5' -> 3')	GGGAATGTCCTCTGCGATGAC	21
			Reverse (5' -> 3')	GAAGGGGATCTCGGGGTTG	19
Acan	11595	NM_007424	Forward (5' -> 3')	GTGGAGCCGTGTTTCCAAG	19
			Reverse (5' -> 3')	AGATGCTGTTGACTCGAACCT	21
Adamts5	23794	NM_011782	Forward (5' -> 3')	GGAGCGAGGCCATTTACAAC	20
			Reverse (5' -> 3')	CGTAGACAAGGTAGCCCACTTT	22
iNOS	18126	NM_010927	Forward (5' -> 3')	GGAGTGACGGCAAACATGACT	21
			Reverse (5' -> 3')	TCGATGCACAACTGGGTGAAC	21
COX2	19225	NM_011198	Forward (5' -> 3')	TGCACTATGGTTACAAAAGCTGG	23
			Reverse (5' -> 3')	TCAGGAAGCTCCTTATTTCCCTT	23

Supplementary Table 3. Genotype of STR and amelogenin loci of ATDC5 cell lines

Number	Marker	Product 1	Product 2	Product 3
1	4-2	229.92		
2	5-5	335.89		
3	6-4	300.46		
4	6-7	334.71		
5	9-2	225.81		
6	12-1	217.84	230.58	
7	15-3	193.58	197.5	
8	18-3	161.1		
9	X-1	404.85		
10	D4S2408			
