# Supplementary material for: An injectable liposome-anchored teriparatide incorporated gallic acid-grafted gelatin hydrogel for osteoarthritis treatment
Source: Nat Commun. 2023 May 31;14:3159. doi: 10.1038/s41467-023-38597-0 (PMC10232438; doi:10.1038/s41467-023-38597-0)
Supplement: Supplementary file 3 — Reporting Summary [file 41467_2023_38597_MOESM3_ESM.pdf]

nature portfolio

Corresponding author(s): Peng Liu, Yingqi Chen, Hui Zeng.

Last updated by author(s): Apr 29, 2023

Reporting Summary

Nature Portfolio wishes to improve the reproducibility of the work that we publish. This form provides structure for consistency and transparency in reporting. For further information on Nature Portfolio policies, see our Editorial Policies and the Editorial Policy Checklist.

Statistics

For all statistical analyses, confirm that the following items are present in the figure legend, table legend, main text, or Methods section.

n/a	Confirmed	
☐	☒	The exact sample size (*n*) for each experimental group/condition, given as a discrete number and unit of measurement
☐	☒	A statement on whether measurements were taken from distinct samples or whether the same sample was measured repeatedly
☐	☒	The statistical test(s) used AND whether they are one- or two-sided<br>*Only common tests should be described solely by name; describe more complex techniques in the Methods section.*
☒	☐	A description of all covariates tested
☐	☒	A description of any assumptions or corrections, such as tests of normality and adjustment for multiple comparisons
☐	☒	A full description of the statistical parameters including central tendency (e.g. means) or other basic estimates (e.g. regression coefficient) AND variation (e.g. standard deviation) or associated estimates of uncertainty (e.g. confidence intervals)
☐	☒	For null hypothesis testing, the test statistic (e.g. *F*, *t*, *r*) with confidence intervals, effect sizes, degrees of freedom and *P* value noted<br>*Give P values as exact values whenever suitable.*
☒	☐	For Bayesian analysis, information on the choice of priors and Markov chain Monte Carlo settings
☒	☐	For hierarchical and complex designs, identification of the appropriate level for tests and full reporting of outcomes
☒	☐	Estimates of effect sizes (e.g. Cohen's *d*, Pearson's *r*), indicating how they were calculated

Our web collection on statistics for biologists contains articles on many of the points above.

Software and code

Policy information about availability of computer code

Data collection	ImageJ software (version 1.53a), FlowJo software (version 10.4), GraphPad Prism (Version 9.0.2).
Data analysis	GraphPad Prism (Version 9.0.2).

For manuscripts utilizing custom algorithms or software that are central to the research but not yet described in published literature, software must be made available to editors and reviewers. We strongly encourage code deposition in a community repository (e.g. GitHub). See the Nature Portfolio guidelines for submitting code & software for further information.

Data

Policy information about availability of data

All manuscripts must include a data availability statement. This statement should provide the following information, where applicable:

- Accession codes, unique identifiers, or web links for publicly available datasets
- A description of any restrictions on data availability
- For clinical datasets or third party data, please ensure that the statement adheres to our policy

All the data of this study are available within the article and Supplementary Information files or from the corresponding author on request. Source data are provided with this paper. Source data are available at https://doi.org/10.6084/m9.figshare.22634641.

Human research participants

Policy information about studies involving human research participants and Sex and Gender in Research.

Reporting on sex and gender	Not applicable.
Population characteristics	Not applicable.
Recruitment	Not applicable.
Ethics oversight	Not applicable.

Note that full information on the approval of the study protocol must also be provided in the manuscript.

Field-specific reporting

Please select the one below that is the best fit for your research. If you are not sure, read the appropriate sections before making your selection.

☒ Life sciences ☐ Behavioural & social sciences ☐ Ecological, evolutionary & environmental sciences

For a reference copy of the document with all sections, see nature.com/documents/nr-reporting-summary-flat.pdf

Life sciences study design

All studies must disclose on these points even when the disclosure is negative.

Sample size	1. Mice and surgical procedures: Female C57BL/6J mice, 8-week-old mice, (of 40 mice in all), with 8 mice per group and five groups of all. For this part, the significant level was set as 0.05, the power was set as 0.8, the differences was set as 0.8, the sample size was calculated as no less than 6, and therefore, 8 mice per group was investigated.<br>2. In vivo retention of hydrogel in arthrography: Female C57BL/6J mice, 8-week-old mice, (of 9 mice in all) with 3 mice per group and three groups of all. In our preliminary study, we treat the mice with hydrogel via intra-articularly and the hydrogel could be formed in situ stablely. For this part, the significant level was set as 0.05, the power was set as 0.8, the differences was set as 3, the sample size was calculated as 3 mice per group.<br>3. The in situ colloidal formation of hydrogel in vivo: Female C57BL/6J mice, 8-week-old mice, (of 6 mice in all), with 3 mice per group and two groups of all. Similarly, we treat the mice with hydrogel via intra-articularly and the hydrogel could be formed in situ stablely in our preliminary study,. For this part, the significant level was set as 0.05, the power was set as 0.8, the differences was set as 3, the sample size was calculated as 3 mice per group.<br>4. Gait analysis and the ROM detection of mice after intra-articular injection with GLP hydrogel: Female C57BL/6J mice, 8-week-old mice, (of 15 mice in all), with 3 mice per group and five groups of all. Similarly, we treat the mice with hydrogel via intra-articularly and the hydrogel could be formed in situ stablely in our preliminary study,. For this part, the significant level was set as 0.05, the power was set as 0.8, the differences was set as 3, the sample size was calculated as 3 mice per group. Moreover, we have refered to some published articles as well.
Data exclusions	Not applicable.
Replication	All experiments were performed biologically and independently in triplicates at least and all attempts at replication were successful.
Randomization	The mice were divided randomly according to the complete random numbers, which was performed as the following steps.<br>1. Number the mice: every mouse got a number from 1 to 40 when thay were purchased into laboratory;<br>2. Obtain the random numbers: Assign different random numbers to all 40 animals in the Excle table;<br>3. Arrange random numbers: rearrange animal numbers in the ascending order of the attached random numbers;<br>4. Sequential grouping: every five mouse was divided into 1, 2, 3, 4 and 5 groups in sequence. Finally, 8 mice were allocated into experimental groups randomly.
Blinding	The relevant evaluation such as histological change were evaluated blindly by two researchers.<br>Apart from the histology evaluation, the investigators were blinded to group allocation during data collection and analysis.

Reporting for specific materials, systems and methods

We require information from authors about some types of materials, experimental systems and methods used in many studies. Here, indicate whether each material, system or method listed is relevant to your study. If you are not sure if a list item applies to your research, read the appropriate section before selecting a response.

Materials & experimental systems

n/a	Involved in the study
☐	☒ Antibodies
☐	☒ Eukaryotic cell lines
☒	☐ Palaeontology and archaeology
☐	☒ Animals and other organisms
☒	☐ Clinical data
☒	☐ Dual use research of concern

Methods

n/a	Involved in the study
☒	☐ ChIP-seq
☐	☒ Flow cytometry
☒	☐ MRI-based neuroimaging

Antibodies

Antibodies used

IF: Ki-67 (Cat#AF1738, Ki67 Rabbit Monoclonal Antibody, 1:200, Beyotime Biotechnology, China), c-Fos (Cat#ab222699, Anti-c-Fos Monoclonal antibody, 1:1000, abcam, UK), PTH1R (Cat#BS2710, PTH/PTHrP-R (L187) polyclonal antibody, 1:200, Bioword Technology, US), SOX9 (Cat#ab185966, Anti-SOX9 antibody, 1:200, abcam, UK), Bcl-2 (Cat#AF6285, Bcl2 Rabbit Polyclonal Antibody, 1:100, Beyotime Biotechnology, China), BAX (Cat#AF0057, BAX Rabbit Polyclonal Antibody, 1:200, Beyotime Biotechnology, China), and Goat Anti-Rabbit IgG (H&L) (Cat#ab150077, Alexa Fluor® 488, 1:1000, abcam, UK).
Western Blot: PTH1R (Cat#BS2710, PTH/PTHrP-R (L187) polyclonal antibody, 1:1000, Bioword Technology, US), SOX9 (Cat#ab185966, Anti-SOX9 antibody, 1:5000, abcam, UK), MMP13 (Cat#ab84594, Anti-MMP13 antibody, 1:1000, abcam, UK), ADAMTS5 (Cat#ab41037, Anti-ADAMTS5 antibody, 1:250, abcam, UK), p-PI3K (Cat#17366, Phospho-PI3 Kinase p85 (Tyr199) (E3U1H) Rabbit (Tyr458)/p55 mAb, 1:1000, CST, US), PI3K (Cat#ab191606, Anti-PI 3 Kinase p85 alpha antibody, 1:1000, abcam, UK), p-AKT (Cat#4060, Phospho-Akt (Ser473) (D9E) XP® Rabbit mAb, 1:1000, CST, US), AKT (Cat#4691, Akt (pan) (C67E7) Rabbit mAb, 1:1000, CST, US), Gapdh (Cat#AC002, GAPDH Mouse mAb, 1:5000, ABclonal, China), and HRP Goat Anti-Mouse IgG (H+L) antibody (Cat#AS003, 1:5000, ABclonal, China).
IHC PTH1R (Cat#BS2710, PTH/PTHrP-R (L187) polyclonal antibody, 1:400, Bioword Technology, US), SOX9 (Cat#ab185966, Anti-SOX9 antibody, 1:1000, abcam, UK), ACAN (Cat#13380-1-AP, Aggrecan Polyclonal antibody, 1:500, Proteintech, China), COLIIA1 (Cat#28459-1-AP, Collagen Type II Polyclonal antibody, 1:500, Proteintech, China), MMP13 (Cat#18165-1-AP, MMP13 Polyclonal antibody, 1:500, Proteintech, China); ADAMTS5 (Cat#bs-3573R, Rabbit Anti-ADAMTS5 Polyclonal Antibody, 1:200, Bioss, China), Bcl-2 (Cat#68103-1-Ig, Bcl2 Monoclonal antibody, 1:500, Proteintech, China) and BAX (Cat#AF0057, BAX Rabbit Polyclonal Antibody, 1:200, Beyotime Biotechnology, China).

Validation

IF: Ki-67 (Cat#AF1738, Ki67 Rabbit Monoclonal Antibody, 1:200, Beyotime Biotechnology, China), c-Fos (Cat#ab222699, Anti-c-Fos Monoclonal antibody, mouse, 1:1000, abcam, UK), PTH1R (Cat#BS2710, PTH/PTHrP-R (L187) polyclonal antibody, mouse, 1:200, Bioword Technology, US), SOX9 (Cat#ab185966, Anti-SOX9 antibody, mouse, 1:200, abcam, UK), Bcl-2 (Cat#AF6285, Bcl2 Rabbit Polyclonal Antibody, mouse, 1:100, Beyotime Biotechnology, China), BAX (Cat#AF0057, BAX Rabbit Polyclonal Antibody, mouse, 1:200, Beyotime Biotechnology, China), and Goat Anti-Rabbit IgG (H&L) (Cat#ab150077, Alexa Fluor® 488, mouse, 1:1000, abcam, UK).
Western Blot: PTH1R (Cat#BS2710, PTH/PTHrP-R (L187) polyclonal antibody, mouse, 1:1000, Bioword Technology, US), SOX9 (Cat#ab185966, Anti-SOX9 antibody, mouse, 1:5000, abcam, UK), MMP13 (Cat#ab84594, Anti-MMP13 antibody, mouse, 1:1000, abcam, UK), ADAMTS5 (Cat#ab41037, Anti-ADAMTS5 antibody, mouse, 1:250, abcam, UK), p-PI3K (Cat#17366, Phospho-PI3 Kinase p85 (Tyr199) (E3U1H) Rabbit (Tyr458)/p55 mAb, mouse, 1:1000, CST, US), PI3K (Cat#ab191606, Anti-PI 3 Kinase p85 alpha antibody, mouse, 1:1000, abcam, UK), p-AKT (Cat#4060, Phospho-Akt (Ser473) (D9E) XP® Rabbit mAb, mouse, 1:1000, CST, US), AKT (Cat#4691, Akt (pan) (C67E7) Rabbit mAb, mouse, 1:1000, CST, US), Gapdh (Cat#AC002, GAPDH Mouse mAb, mouse, 1:5000, ABclonal, China), and HRP Goat Anti-Mouse IgG (H+L) antibody (Cat#AS003, mouse, 1:5000, ABclonal, China).
IHC PTH1R (Cat#BS2710, PTH/PTHrP-R (L187) polyclonal antibody, mouse, 1:400, Bioword Technology, US), SOX9 (Cat#ab185966, Anti-SOX9 antibody, mouse, 1:1000, abcam, UK), ACAN (Cat#13380-1-AP, Aggrecan Polyclonal antibody, mouse, 1:500, Proteintech, China), COLIIA1 (Cat#28459-1-AP, Collagen Type II Polyclonal antibody, mouse, 1:500, Proteintech, China), MMP13 (Cat#18165-1-AP, MMP13 Polyclonal antibody, mouse, 1:500, Proteintech, China); ADAMTS5 (Cat#bs-3573R, Rabbit Anti-ADAMTS5 Polyclonal Antibody, mouse, 1:200, Bioss, China), Bcl-2 (Cat#68103-1-Ig, Bcl2 Monoclonal antibody, mouse, 1:500, Proteintech, China) and BAX (Cat#AF0057, BAX Rabbit Polyclonal Antibody, mouse, 1:200, Beyotime Biotechnology, China).

Eukaryotic cell lines

Policy information about cell lines and Sex and Gender in Research

Cell line source(s)

The ATDC5 chondrogenic cell lines were purchased from KeyGEN BioTECH (Cat#KG445, KeyGEN, China) and cultured in DMEM/F12 (Gibico, US) with 5% FBS (Gibico, US), 1% penicillin and streptomycin (Gibico, US) in a humidified incubator (5% CO_2, 37 °C). In addition, the ATDC5 cells are stimulated with insulin (10 μg/ml) according to the instructions for further study.

Authentication

Via the microscope system.

Mycoplasma contamination

We confirmed that the cell line tested negative for mycoplasma contamination during the culture.

Commonly misidentified lines
(See ICLAC register)

Not applicable.

Animals and other research organisms

Policy information about studies involving animals; ARRIVE guidelines recommended for reporting animal research, and Sex and Gender in Research

Laboratory animals	All mice (female, 8-week-old) were purchased from Shanghai Model Organisms (Shanghai, China) and housed in Topbio-technology (Shenzhen, China) under standard laboratory conditions (12 hours light/dark cycle, ambient temperature (25°C), and humidity (50%)) with food and water freely.
Wild animals	Not applicable.
Reporting on sex	Female.
Field-collected samples	Not applicable.
Ethics oversight	All the animal procedures were conducted following the instructions approved by the Institutional Animal Care and Use Committee (IACUC) of Peking University Shenzhen Hospital. All the animal study used this kind of mice and the animal treatment process was followed this IACUC protocol.

Note that full information on the approval of the study protocol must also be provided in the manuscript.

Flow Cytometry

Plots

Confirm that:

☒ The axis labels state the marker and fluorochrome used (e.g. CD4-FITC).

☒ The axis scales are clearly visible. Include numbers along axes only for bottom left plot of group (a 'group' is an analysis of identical markers).

☒ All plots are contour plots with outliers or pseudocolor plots.

☒ A numerical value for number of cells or percentage (with statistics) is provided.

Methodology

Sample preparation	For FCM tests, the ATDC5 cells were cultured with the GGA hydrogel and GLP hydrogel for 48 h by means of the same cell culture method as aforementioned, and then the cells were digested with trypsin. The cells were stained with the Cell Cycle Staining Kit (Cat#CCS012, Multi Sciences, China) following the instructions, and then the cells were measured with FCM according to the protocol.<br><br>The same cell treatment process and culture method were utilized for further FCM assessment. The Annexin V-FITC/PI apoptosis kit (Cat#AT101C, Multi Sciences, China) was used and the procedures of FCM assessment were the same as aforementioned.
Instrument	Beckman Coulter CytoFlex LX.
Software	Flowjo software (version 10.4).
Cell population abundance	30000 cells/ml.

Gating strategy

For the part of the apoptosis evaluation:
1. Original data from samples were analyzed in Flowjo, Cell gating in FSC-a(x axis) and ssc-a(y axis) showed general cell size to discard cell fragments.
2. Double click on the analyzed cell population in the SSC/FSC diagram and select FITC on the X-axis to indicate AnnexinV-FITC. The Y-axis is PE-Texas, which is used to represent PI.
3. Living cells and apoptotic cells were defined by a four-point gate. The four-point gate should define the negative confidence area according to the negative control. According to the trend of cell grouping, the demarcation line of the four-point gate can be located at the cluster of negative and positive cells, because normal cultured cells will also undergo natural apoptosis, allowing negative control non-specific fluorescence signal (false positive) is generally less than 1%-3%.
4. Q1: (AnnexinV-FITC) -/PI+, the cells in this region are necrotic cells. There may also be a small number of late apoptotic cells, or even mechanically damaged cells. Q2: (AnnexinV+FITC) -/PI+. Cells in this region are late apoptotic cells. Q3: (AnnexinV-FITC) +/PI-, cells in this region are early apoptotic cells. Q4: (AnnexinV-FITC) -/PI-, cells in this region are living cells.

For the part of the cellcycle evaluation:
1. Original data from samples were analyzed in Flowjo,, double-click to open the original data, select the FSC-A for the X-axis while the SSC-A for the Y-axis, an irregular loop gate was selected to circle the cells that fit the analysis conditions.
2. Double-click the cell mass to enter a new interface.
3. Circle the single cells with a framed door. Return to the home screen, right-click single cells, and select cell cycle.
4. Go to the result analysis screen and select channel PE-A. Return to the main screen, drag cell cycle directly into the right dialog box, and save it to the required file format.

☒ Tick this box to confirm that a figure exemplifying the gating strategy is provided in the Supplementary Information.
