# Supplementary material for: An injectable liposome-anchored teriparatide incorporated gallic acid-grafted gelatin hydrogel for osteoarthritis treatment
Source: Nat Commun. 2023 May 31;14:3159. doi: 10.1038/s41467-023-38597-0 (PMC10232438; doi:10.1038/s41467-023-38597-0)
Supplement: Supplementary file 4 — Source data [file 41467_2023_38597_MOESM4_ESM.zip › Dataset 2 Source Data file Fig.5m.pdf]

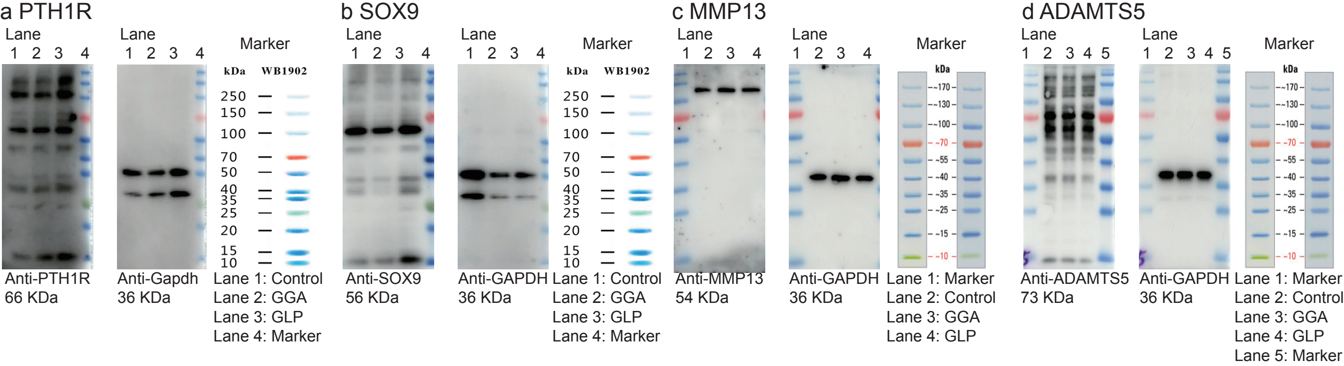
a PTH1R
Lane
1 2 3 4
Lane
1 2 3 4
Marker
kDa WB1902
250
150
100
70
50
40
35
25
20
15
10
Anti-PTH1R
66 KDa
Anti-Gapdh
36 KDa
Lane 1: Control
Lane 2: GGA
Lane 3: GLP
Lane 4: Marker
b SOX9
Lane
1 2 3 4
Lane
1 2 3 4
Marker
kDa WB1902
250
150
100
70
50
40
35
25
20
15
10
Anti-SOX9
56 KDa
Anti-GAPDH
36 KDa
Lane 1: Control
Lane 2: GGA
Lane 3: GLP
Lane 4: Marker
c MMP13
Lane
1 2 3 4
Lane
1 2 3 4
Marker
kDa
~170
~130
~100
~70
~55
~40
~35
~25
~15
~10
Anti-MMP13
54 KDa
Anti-GAPDH
36 KDa
Lane 1: Marker
Lane 2: Control
Lane 3: GGA
Lane 4: GLP
d ADAMTS5
Lane
1 2 3 4 5
Lane
1 2 3 4 5
Marker
kDa
~170
~130
~100
~70
~55
~40
~35
~25
~15
~10
Anti-ADAMTS5
73 KDa
Anti-GAPDH
36 KDa
Lane 1: Marker
Lane 2: Control
Lane 3: GGA
Lane 4: GLP
Lane 5: Marker
