# Supplementary figures and images for: An injectable liposome-anchored teriparatide incorporated gallic acid-grafted gelatin hydrogel for osteoarthritis treatment
Source: Nat Commun. 2023 May 31;14:3159. doi: 10.1038/s41467-023-38597-0 (PMC10232438; doi:10.1038/s41467-023-38597-0)

## a p-PI3K

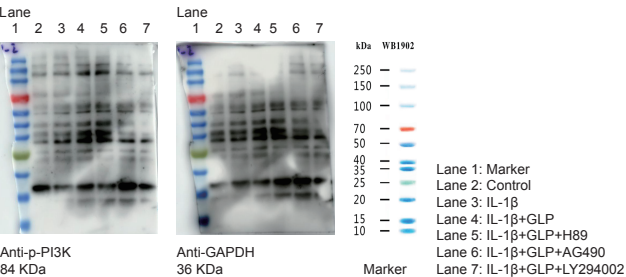

## c p-AKT

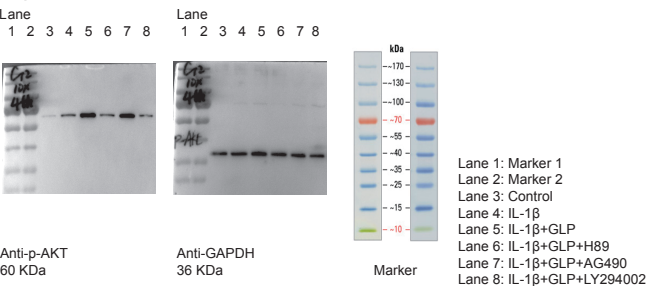

## e ADAMTS5

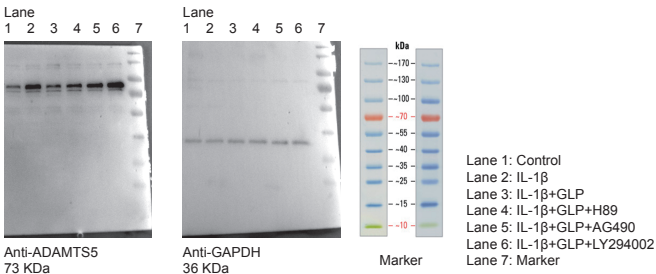

## b PI3K

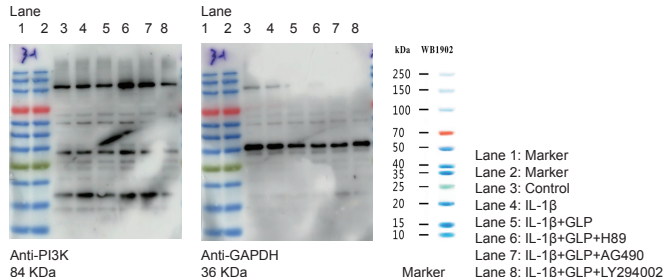

## d AKT

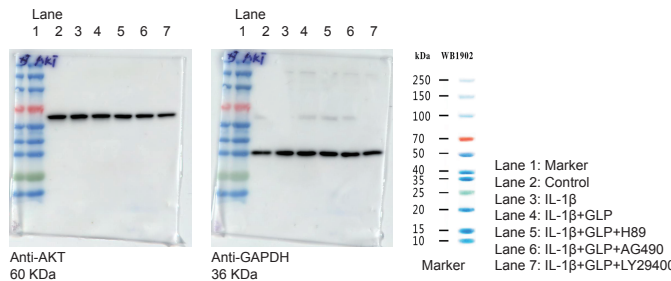

Supplement: Supplementary file 4 — Source data [file 41467_2023_38597_MOESM4_ESM.zip › Dataset 3 Source Data file Fig.6q.pdf]
